# Pathological findings of pulmonary papillary adenoma with EGFR mutation and literature review: two cases report

**DOI:** 10.1186/s13019-024-02852-2

**Published:** 2024-06-20

**Authors:** Lu Huang, Yi Liu, Changjun Yi, Lin Han, Shuang Li, Xingzi Li, Xuehui Wu

**Affiliations:** 1 Department of Pathology, The First College of Clinical Medical Science, China Three Gorges University, Yichang, 443000 China; 2 Department of Pathology, Yichang Central People’s Hospital, Yichang, 443100 China; 3 Third-Grade Pharmacological Laboratory On Traditional Chinese Medicine, State Administration of Traditional Chinese Medicine, China Three Gorges University, Yichang, 443002 China; 4 Department of Pathology, Yiling Hospital, Yichang, 443100 China; 5 Yichang Ackerman Pathological Diagnostic Centre, Yichang, 443000 China

**Keywords:** Lung, Papillary adenoma, EGFR mutation, Clinicopathological features

## Abstract

**Objective:**

Pulmonary papillary adenoma is an extremely rare benign tumor. It is derived from type II lung cells and club cells, suggesting that it may originate from stem cells with two-way differentiation. Only one case has been reported with FGFR2-IIIb overexpression.

**Methods:**

Two cases of pulmonary papillary adenoma with available data on clinical features, histological morphology, immunophenotype and molecular characteristics were analyzed.

**Results:**

Both tumors were well-circumscribed unencapsulated nodules composed of papillary structures with fibrovascular cores lined by a single layer of cuboidal or columnar epithelium without necrosis, nuclear atypia and mitoses, or invasion. But malignant transformation features include complex branching structures and significantly enlarged, irregular, and crowded malignant cells in one case. Immunohistochemistry showed that the tumor cells were strongly positive for TTF1, NapsinA, EMA and CK7 and negative for CEA and P63, with a low Ki-67 proliferation index. The EGFR somatic mutation exon19:c.2236_2256delinsATC (p.E746_S752delinsI) was found in one case by next-generation sequencing (NGS) technology.

**Conclusion:**

Pulmonary papillary adenoma is very rare. Virtually all papillary adenomas are clinically silent and discovered incidentally. They are benign tumors, and resection is curative. An EGFR 19 exon deletion mutation in a patient with this tumor type was detected for the first time by NGS, and our results suggest that the malignant transformation of pulmonary papillary adenoma may be mediated by EGFR mutation.

Papillary adenoma of the lung is a rare benign tumor originating from the alveolar epithelium. Almost 30 cases have been reported, with a male predominance and an age range of 2 months to 70 years (median age: 34 years) [1, 2]. Most patients with pulmonary papillary adenoma are asymptomatic, and a few show nonspecific symptoms such as cough, sputum, hemoptysis, chest pain, etc. CT typically shows well circumscribed tumors (2–60 mm in diameter), and the tumors usually occur in the peripheral lung, especially in the lower lobe of the left lung [3]. The genetic characteristics of pulmonary papillary adenoma are rarely reported, and one case with FGFR2-IIIb mRNA overexpression by PCR has been reported [4]. Thus, clarification of the genetic characteristics of these tumors is crucial to prevent their progression and facilitate treatment. We present two cases of pulmonary papillary adenoma and the corresponding clinicopathological characteristics and report the first instance of EGFR 19 exon mutation in this tumor type.

## Materials and Methods

Tissue specimens were fixed in 10% formalin, routinely embedded in paraffin, sectioned (3 μm thick), and stained with hematoxylin eosin for histological examination. Immunohistochemical staining was performed on 3 µm sections, and the primary antibodies included TTF1, NapsinA, EMA, CK7, TG, P63, CEA, P53, C-myc, CD34, D2-40, S100 and Ki-67. All primary antibodies and PV6000 kits were provided by Beijing ZSGB Biotechnology Co. All antigens were retrieved with citric acid (pH 6.0) or EDTA (pH 9.0) heated by a pressure cooker and detected by PV-6000 staining, and the procedures were carried out in accordance with each antibody IHC protocol. Next-generation sequencing technology was used to assess the status of 22 lung cancer-related genes (AKT1, ALK, BRAF, CDKN2A, DDR2, EGFR, ERBB2, FGFR1, KRAS, MAP2K1, MET, NF1, NRAS, NTRK1, NTRK2, NTRK3, PIK3CA, PTEN, RET, ROS1 STK11 and TP53) in paraffin-embedded specimens at the Shanghai Ackerman Diagnostic Pathology Center.

## Results

### Clinical summary

**Fig. 1:**
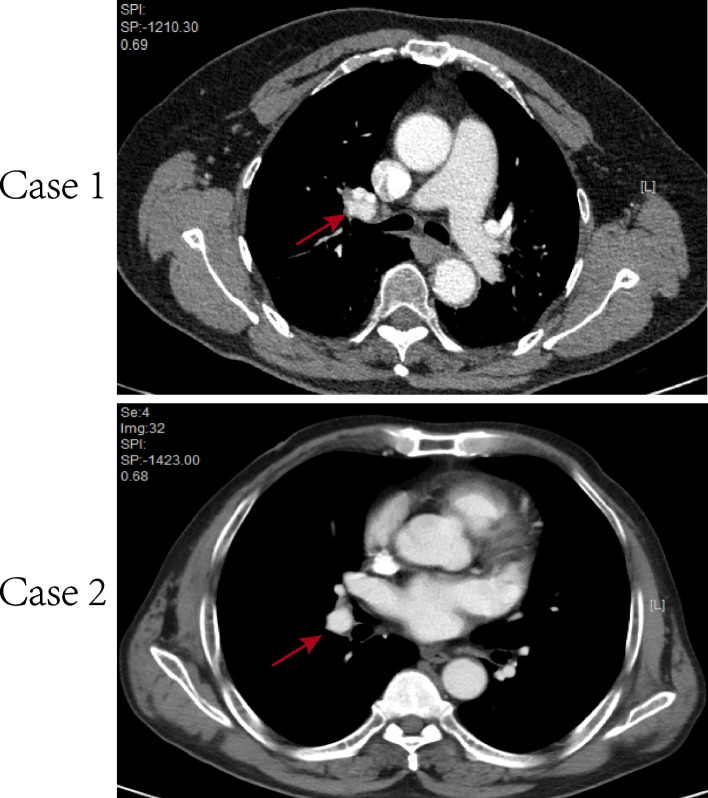
Imaging characteristics of two patients. **A**,** B** CT showed a well-defined nodule in the lower lobe of the right lung, measuring approximately 3.5 × 3.3 × 2.8 cm and 4.4 × 4.1 × 3.5 cm

There were two patients with pulmonary papillary adenoma: one was a 58-year-old female with epigastric pain, and the other was a 57-year-old male with intermittent dizziness and headache. The female patient had no history of smoking, and the male patient had a history of smoking (20 years: 10 pcs/day), but neither of them had any related occupational exposure. Neither had obvious respiratory symptoms, such as cough, sputum, hemoptysis or chest pain, and the tumors were incidentally detected on radiological examination. Each patient had a nodule in the lower lobe of the right lung (Fig. 1). They both underwent complete resection of the lesion with no recurrence or signs of metastasis for 12 and 18 months, respectively. The clinical characteristics of both patients are shown in Table 1.

**Table 1:** The clinical characteristics of both patients

Num	Sex/Age	Smoking history	Comorbidity	Respiratory symptoms	CT chest performance	CEA	Neuron specific enolase	Treatment	Follow-up
Case 1	F/58	NO	Hypertension; Fatty liver; Kidney Stones	NO	GGO (LUL) (3.5 × 3.3 × 2.8)	0.49	8.3 (0–16.3)	Lung lobectomy	Good
Case 2	M/57	20 years; 10 pcs/day	Hypertension; Cerebral infarction match	NO	GGO (LUL) (4.4 × 4.1 × 3.5)	/	18.93	Lung lobectomy	Good

### Histological examination

#### Gross appearance

The specimens of both cases were obtained by right lower lobe resection, measuring 11 × 5.5 × 3.8 cm and 15 × 11 × 4.5 cm; each had a clear boundary around a grayish-white nodule, and the nodules measured approximately 3.5 × 3.3 × 2.8 cm and 4.4 × 4.1 × 3.5 cm.

#### Microscopic examination

**Fig. 2:**
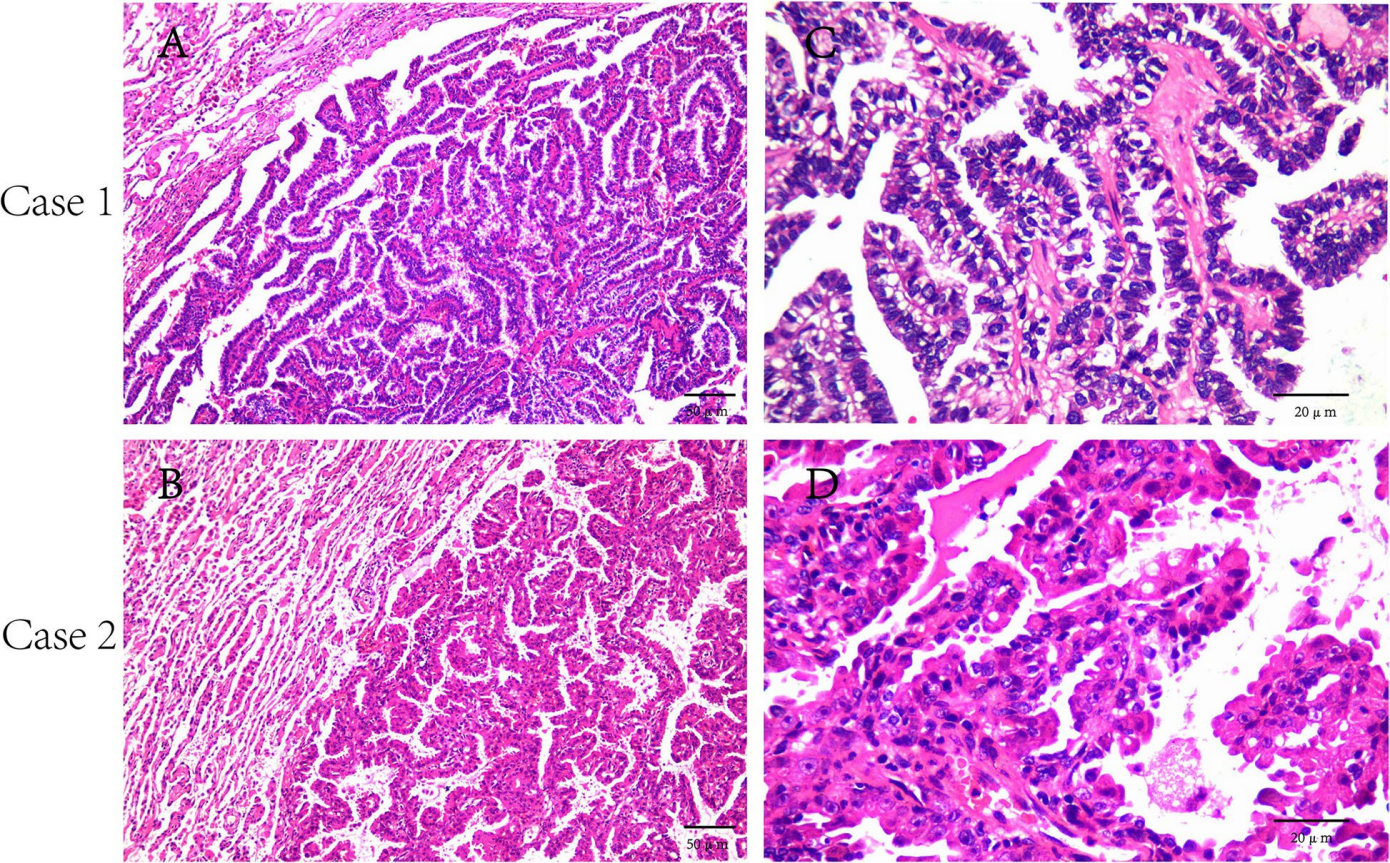
H&E staining of pulmonary papillary adenoma. **A**, **B** Both of the tumor tissues were clearly demarcated from the surrounding normal alveolar tissues without fibrous septa. Scale Bar, 50 μm. **C**, **D** The papillae were lined with single-layered or pseudo-layered cuboidal or columnar tumor cells, with uniform cell size, eosinophilic cytoplasm, round or ovoid nuclei, and inconspicuous nucleoli. Scale Bar, 20 μm

Both of the tumor tissues were clearly demarcated from the surrounding normal alveolar tissues without fibrous septa. The tumor was composed of papillary structures with fibrovascular cores and focal complex branching architecture (Fig. 2 A), and the papillae were lined with single-layered or pseudolayered cuboidal or columnar tumor cells, with significantly enlarged, irregular, and crowded malignant cells, and inconspicuous nucleoli (Fig. 2 C) in case1. The tumors were composed of papillary structures with fibrovascular cores and no complex branching architecture (Fig. 2 B), and the papillae were lined with single-layered or pseudolayered cuboidal or columnar tumor cells, with uniform cell size, eosinophilic cytoplasm, round or ovoid nuclei, and inconspicuous nucleoli (Fig. 2 D) in case2. There was no nuclear schismatic activity and no necrosis or infiltrative growth pattern, with some small lymphocytes infiltrating the interstitium. Therefore, both tumors were diagnosed as pulmonary papillary adenoma, one of which was accompanied by malignant transformation.

### Immunohistochemistry (IHC)

**Fig. 3:**
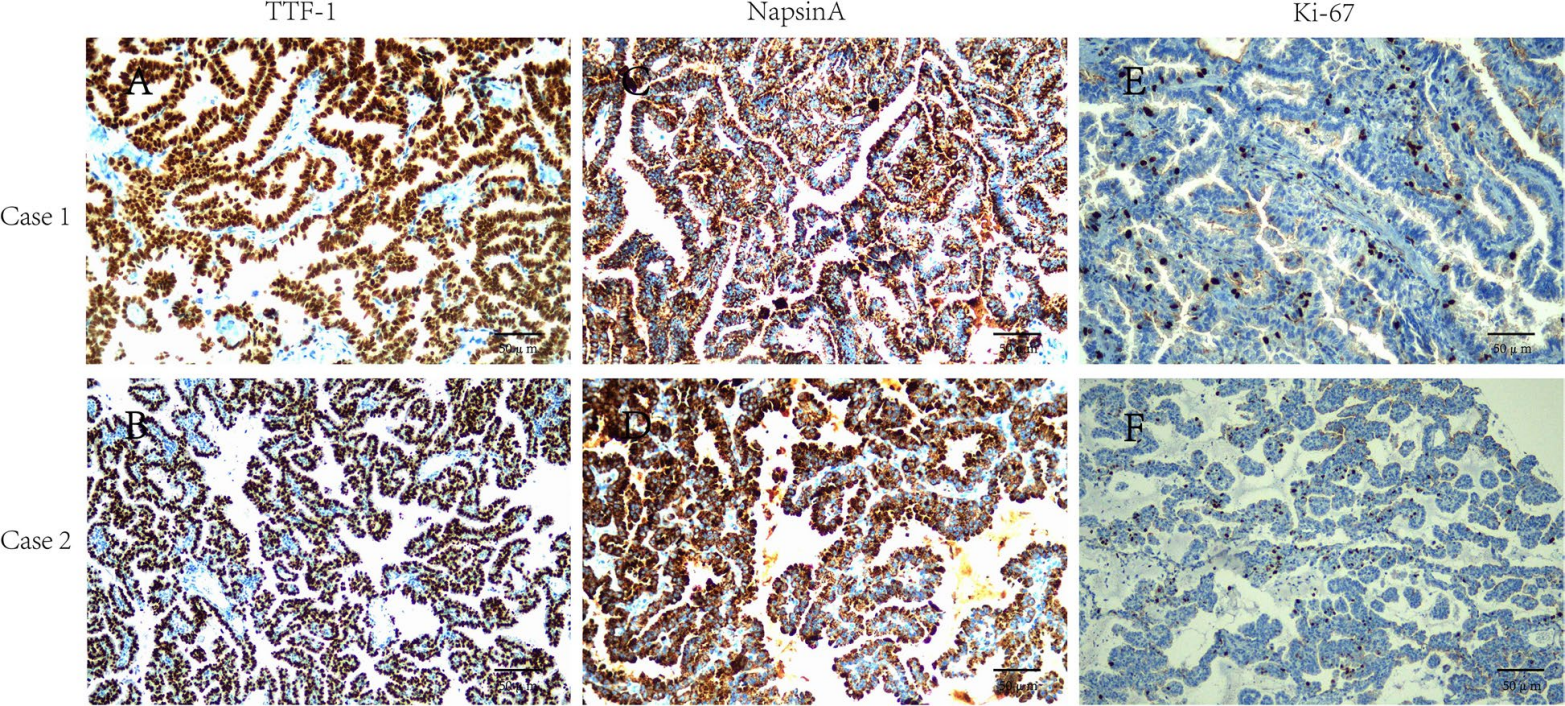
Immunohistochemical expression of TTF1, Napsin A and Ki-67 in pulmonary papillary adenoma. **A**, **B** The tumor cells were diffuse strongly positive for TTF1. Scale Bar, 50 μm. **C**, **D** The tumor cells were diffuse strongly positive for Napsin A. Scale Bar, 50 μm. **E**, **F** The proliferation index KI-67 of pulmonary papillary adenoma cells was less than 5%. Scale Bar, 50 μm

Both of the tumor cells were diffuse and strongly positive for TTF1 (Fig. 3A, B), Napsin A (Fig. 3C, D), EMA and CK7, and the proliferation index KI-67 was less than 5% (Fig. 3E, F), with P53 showing wild-type expression patterns and negative for CEA, C-myc and P63. CD34, D2-40, and S100 were used to confirm that tumor cells had no vascular or nerve invasion.

### NGS technology examination

A total of 22 lung cancer-related genes of the two patients were tested by the Shanghai Ackerman Diagnostic Pathology Center and one case with an EGFR somatic mutation: exon19:c.2236_2256delinsATC (p.E746_S752delinsI) was detected in one case (the female patient), and no somatic mutation was detected in the other (the male patient).

## Discussion

Pulmonary papillary adenoma is a rare tumor that occurs mainly in the peripheral portion of the lung. Most studies have considered that the tumor’s biological behavior is benign and resection is curative, but few studies have shown that this tumor may undergo malignant transformation. Dessy E et al. reported two cases of pulmonary papillary adenomas with aggressive growth behavior [5], with tumor cells present in the alveolar structures and pleura. In another retrospective study of 15 cases of pulmonary papillary adenoma, two had malignant manifestations, including tumor cells of variable size, deep nuclear chromatin, easily visible nuclear schizophrenic images, partial infiltration of tumor cells in the interstitium, and a Ki-67 proliferation index exceeding 25% in the heterogeneous region [3]. The etiology and pathogenesis of pulmonary papillary adenoma are not clear. Some studies have shown by electron microscopy that the intranuclear inclusion bodies in tumor cells show tubular structures arranged in aggregates similar to lamellipodia, indicating that the tumor cells may originate from type II alveolar epithelial cells or club cells [6], suggesting that they may arise from bidirectionally differentiated stem cells.

Histologically, the tumor showed unencapsulated circumscribed peripheral papillary glandular neoplasm with a single layer of cytologically bland cuboidal to columnar cells lining fibrovascular cores, no nuclear atypia, minimal to no mitoses, no necrosis, no complex branching architecture, and absence of invasion. These features make it difficult to diagnose this tumor based on frozen specimens, as it needs to be differentiated from sclerosing pneumocytoma, pulmonary papillary adenocarcinoma, bronchiolar adenoma/ciliated muconodular papillary tumor, alveolar adenoma, and metastatic papillary thyroid carcinoma of the lung. (1) Sclerosing pneumocytoma is composed of two types of cells, including surface cells similar to type II alveolar cells and interstitial polygonal cells as well as a mixture of solid, papillary, spongy angiomatous and sclerotic growth patterns, and its tumor cells are positive for TTF1 and EMA both on the surface and interstitial cells, which can be distinguished from Sclerosing pneumocytoma [7]. (2) Plmonary papillary adenoma is a specific subtype of invasive lung adenocarcinoma that shows indistinct demarcation from the surrounding area and fibrous capsule, histological features of infiltrative growth into the peripheral tissues, a complex branching papillary structure, obvious heterogeneity of the lining tumor cells, multiple nuclear mitosis images, and a high KI67 proliferation index [8]. (3) Bronchial adenoma (ciliated mucinous nodular papillary tumor) is a benign peripheral lung tumor consisting of a bilayer epithelium of the fine bronchial type containing a continuous basal cell layer with clear demarcation between the tumor and surrounding tissues. Immunohistochemistry reveals P63-positive continuous basal layer cells, which can be distinguished from pulmonary papillary adenoma [9]. (4) Alveolar adenoma is a well-defined tumor, usually encapsulated, consisting of multiple cystic cavities mimicking alveolar cavities filled with eosinophilic granular material and lined with a single layer of type II alveolar cells, with characteristic spindle/inflammatory stroma and without papillary morphology [10]. (5) Pulmonary metastatic papillary thyroid carcinoma is clearly demarcated from surrounding tissues and may have a fibrous envelope. The tumor cells show overlapping, crowded, vacuolated, nuclear grooves and pseudoinclusion bodies. Immunohistochemistry revealed that tumor cells were positive for TTF1 and TG [11].

Some research has shown that pulmonary papillary adenoma can have malignant features, including tumor cell dysplasia and short, flat, or tubular nipples [12]. The etiology of pulmonary papillary adenoma is still unknown. In a reported case of pulmonary papillary adenoma in a 16-year-old Japanese female, the expression levels of fibroblast growth factor 10 (FGF10), keratinocyte growth factor (KGF) and fibroblast growth factor receptor 2 IIIb (FGFR2 IIIb) were detected using immunohistochemistry, in situ hybridization and real-time reverse transcription-polymerase chain reaction, and FGFR2 IIIb was found to be overexpressed in the tumor tissue, suggesting that it may play an important role in the process of tumor formation [4]. However, no other mutations in pulmonary papillary adenoma were observed by molecular tests [13, 14].

Epidermal growth factor receptor (EGFR) is a member of receptor tyrosine kinase family I. Upon activation of EGFR induced by binding to extracellular ligands, EGFR can facilitate intracellular signaling through a variety of signaling pathways, including RAS/RAF/MAPK, PI3K/Akt/mTOR, and STAT [15], and activation of these signaling pathways can promote cell proliferation [16, 17]. EGFR mutations are more common in adenocarcinoma than in squamous carcinoma, and 51.4% of advanced lung adenocarcinoma patients in Asia have this mutation [18, 19]; the EGFR variation frequency is higher in women and nonsmoking patients [20, 21]. There are two common hotspot mutations in the structural domain of EGFR kinase: exon 19 deletion and exon 21 L858R mutation; exon 19 deletion variants account for approximately 45–50% of EGFR variants in non-small cell lung cancer [22]. EGFR exon19del is in the αC-helix region, and the 19del mutant protein shortens the upper part to bind to the αC helix and activate the kinase conformation by αC helix rotation; the shortened 19del mutant protein has a more compact structure, so the EGFR kinase is in the highest activity state. In our cases, a female patient was found to have an EGFR 19del mutation, and this patient had no smoking history or occupational exposure. However, the patient in our study was still treated for a benign lesion and had not undergone postoperative radiotherapy or chemotherapy. We followed up with the patient for 2 years and found no recurrence or metastasis. For patients with EGFR-mutated papillary adenomas, further study is needed to determine whether extended resection or postoperative treatments are needed. The detection of EGFR mutations provides important laboratory evidence to further investigate the pathogenesis and treatment of pulmonary papillary adenoma.

According to the 2021 World Health Organization Classification of Thoracic Tumours, pulmonary papillary adenoma is a rare benign tumor reported to exhibit malignant transformation [8, 12]. Histologically, malignant transformation features include complex branching structures and invasive growth within the lesion, as well as benign cells similar to normal alveolar epithelium and significantly enlarged, irregular, and crowded malignant cells in the tumor. We reported 2 cases of pulmonary papillary adenoma, one of which had malignant features and detected EGFR mutation. These findings indicate the process of malignant transformation from papillary adenoma to pure papillary adenocarcinoma. We have not only gained a profound understanding of the morphological features of malignant transformation in pulmonary papillary adenoma, but also clarified its molecular changes, which have important guiding significance for clinical practice. Given the potential for malignant transformation of pulmonary papillary adenoma, more active treatment measures should be taken and clinical follow-up observations should be conducted.

## Conclusion

Pulmonary papillary adenomas are clinically very rare and benign tumors, and surgical resection of lobes or segments of the lung is the treatment of choice. However, some studies have shown the malignant transformation of these tumors. We found that a case with EGFR 19del mutation had focal malignant features, suggesting that this tumor may be a prodromal stage in the development of lung carcinoma, but this hypothesis needs to be confirmed with more cases.

## Data Availability

No datasets were generated or analysed during the current study.
